# Estimation of Donor Renal Function After Living Donor Nephrectomy: The Value of the Toulouse-Rangueil Predictive Model

**DOI:** 10.3389/ti.2023.11393

**Published:** 2023-05-19

**Authors:** Thomas Prudhomme, Mathieu Roumiguie, Marc Olivier Timsit

**Affiliations:** ^1^ Department of Urology and Kidney Transplantation, Toulouse-Rangueil University Hospital, Toulouse, France; ^2^ Center for Research in Transplantation and Translational Immunology, Nantes University, INSERM, UMR 1064, Nantes, France; ^3^ Department of Urology and Transplant Surgery, AP-HP, Necker Hospital and European Hospital Georges Pompidou, Paris, France

**Keywords:** living donor nephrectomy, renal function, external validation, predictive model, concordance

Assessment of renal function after living donor nephrectomy represents a current hot topic in kidney transplantation. It is well known that living donor kidney transplantation (LDKT) is the best treatment for end-stage renal disease (ESRD) patients eligible for transplantations [1]. However, it has been demonstrated that living donors are at an increased risk of chronic kidney disease (CKD) and ESRD comparing to healthy non-donors [2, 3], exposing them to cardiovascular and global morbidity and mortality of CKD [4]. For these reasons, personalized instruments to evaluate post-operative renal function are necessary in order to offer the benefit of LDKT to recipients while controlling the immediate and long-term negative effects of reduced nephron mass for the donor.

Almeida et al. [5], have just published in Transplant International, an external validation of the predictive model to estimate the donor renal function after living donor nephrectomy, developed by our team at the Rangueil University Hospital in Toulouse. They reported a significant correlation (Pearson *r* = 0.67; *p* < 0.001) and concordance (Bland-Altman plot with 95% limits of agreement −21.41 to 26.47 mL/min/1.73 m^2^; *p <* 0.001) between predicted and observed 1-year estimated glomerular filtration rate (eGFR). The area under the receiver operating characteristic curve (AUROC) showed a good discriminative ability of the formula to predict CKD [AUC: 0.83 (CI 95%: 0.78–0.88; *p* < 0.001)].

It is very rewarding to see a confirmation of our previous report regarding this predictive model to estimate the 1-year post-donation eGFR and risk of CKD. First, Benoit et al. [6] retrospectively evaluated our living donor cohort and demonstrated that age and preoperative eGFR were strong independent predictors of postoperative eGFR. A formula, using multiple linear regression model, was designed: postoperative eGFR (CKD-EPI, mL/min/1.73 m^2^) = 31.71 + (0.521 × preoperative eGFR) − (0.314 × age). The internal validation reported an optimal statistical performance with a significant correlation (*r* = 0.65) and an AUROC of the model of 0.83 (CI 95%: 0.72–0.93; *p* < 0.001). Then, this model was externally validated using the Necker Hospital living donor cohort [7]. A significant correlation (Pearson *r* = 0.66; *p* < 0.001) and concordance (Bradley-Blackwood F = 49.189; *p* < 0.001) were reported between predicted and observed 1-year eGFR. The AUROC in this external population confirmed discriminative ability of this model to predict CKD (AUC: 0.86 (CI 95%: 0.82–0.89; *p* < 0.01)). Kullik et al. [8] also externally validated the model and reported a good correlation to the observed 1-year post-donation eGFR.

External validation is necessary to determine predictive model reproducibility and generalizability to different patients [9]. Correlation and concordance are usually degraded by external validations [10], the outcomes reported by Almeida et al. [5] confirmed the effectiveness of the formula to estimate the 1-year post-donation eGFR and risk of CKD. The strength of their study is the evaluation of model performance according to the sex of the donor; they reported similar performance between females and males.

Along with an extension of marginal deceased donors (extended criteria donor, donation after circulatory death donors) [11, 12], extending the age limit for living donors to expand the pool has been considered [13, 14]. However, donor age was to be a strong predictor of CKD after living donor nephrectomy which may defer the wish to extend the age limit of donors [6, 15]. Preoperative eGFR was also an independent predictor of postoperative eGFR [6]. Thus, a particular attention should be paid to preoperative eGFR and the risk of postoperative CKD with potential an increasing pool of donor over 60 years. However, considering the performance of the predictive model, the use of this tool at the living donor evaluation consultation, combined with a global donor assessment (i.e., comorbidities assessment, donor age and expected lifespan), could contraindicated some potential donors with predicted lower 1-year eGFR.

The prediction of the postoperative renal function is the key point of donor’s candidate evaluation. This predictive model, represents a simple, low cost, non-invasive tool that can be easily joined to the living donor evaluation routine consultation, paving the way for an improved decision-making process.

## Data Availability

The original contributions presented in the study are included in the article/supplementary material, further inquiries can be directed to the corresponding author.
